# Insights into the genetic architecture of cerebellar lobules derived from the UK Biobank

**DOI:** 10.1038/s41598-024-59699-9

**Published:** 2024-04-25

**Authors:** Amaia Carrión-Castillo, Cedric Boeckx

**Affiliations:** 1 Basque Center on Cognition, Brain and Language (BCBL), Donostia-San Sebastián, Spain; 2 Ikerbasque, Basque Foundation for Science, Bilbao, Spain; 3 Universitat de Barcelona, Barcelona, Spain; 4 Universitat de Barcelona Institute of Complex Systems, Barcelona, Spain; 5 Universitat de Barcelona Institute of Neurosciences, Barcelona, Spain; 6 Catalan Institute for Research and Advanced Studies (ICREA), Barcelona, Spain

**Keywords:** Genetics, Neuroscience

## Abstract

In this work we endeavor to further understand the genetic architecture of the cerebellum by examining the genetic underpinnings of the different cerebellar lob(ul)es, identifying their genetic relation to cortical and subcortical regions, as well as to psychiatric disorders, as well as traces of their evolutionary trajectories. We confirm the moderate heritability of cerebellar volumes, and reveal genetic clustering and variability across their different substructures, which warranted a detailed analysis using this higher structural resolution. We replicated known genetic correlations with several subcortical volumes, and report new cortico-cerebellar genetic correlations, including negative genetic correlations between anterior cerebellar lobules and cingulate, and positive ones between lateral Crus I and lobule VI with cortical measures in the fusiform region. Heritability partitioning for evolutionary annotations highlighted that the vermis of Crus II has depleted heritability in genomic regions of “archaic introgression deserts”, but no enrichment/depletion of heritability in any other cerebellar regions. Taken together, these findings reveal novel insights into the genetic underpinnings of the different cerebellar lobules.

## Introduction

There is ever-increasing evidence for the role of the cerebellum in cognitive processes, extending its functions beyond its classical implication in movement coordination and balance^1,2^. Although the cerebral neocortex continues to be treated as the seat of our “higher-order” cognitive abilities, it is clear that the cerebellum also plays a key role, likely due to its extensive connections with virtually every part of the neocortex (and beyond)^3^, as well as its actual surface area^4^.

Like the cerebral neocortex, the cerebellum consists of multiple areas that exhibit a complex range of connections with other brain regions^5,6^, and is implicated in a wide range of processes, such as sensorimotor, cognitive and social/affective^7–11^. While the sensorimotor cerebellum is represented mostly in the anterior cerebellar lobe (lobules I to V), with lesions of these areas leading to motor disorders (e.g. cerebellar motor syndrome of ataxia, dysmetria), the posterior portions of the cerebellum (lobules VI–IX, Crus I and Crus II) have been implicated in “higher” cognitive processes, with lesions resulting in the cerebellar cognitive affective syndrome^12^. Consequently, the cerebellum (especially the phylogenetically more recent, posterior lobes) has been implicated in a wide range of psychiatric disorders^13,14^. There is also mounting evidence for the evolutionary relevance of cerebellar expansion in the hominin lineage^15–19^.

Two recent studies have investigated the genetics of cerebellar volume and have established that it is a heritable structure, identified tens of associated genetic loci, and revealed genetic links with mental disorders^20,21^. A genetic analysis of cerebellar white-matter microstructure has also identified eleven associated genetic loci and genetic overlap with cognitive and psychiatric disorders^22^. However, these studies focused primarily on the genetic architecture of overall cerebellar volume and cerebellar white matter microstructure. Thus, in this work we take a complementary approach and utilize genetic analyses to examine cerebellar substructures and their genetic underpinnings. We do so by leveraging publicly available GWAS summary statistics for imaging derived phenotypes (IDPs; based on the UK Biobank N $\sim$ 31,000)^23^, as well as on GWASes of other traits such as schizophrenia (SCZ)^24^, autism spectrum disorder (ASD)^25^ and cognitive performance^26^ (Table S1). For this, we mostly rely on secondary analyses of data derived from the UK Biobank dataset, which is a unique resource that enables researchers to examine genetic effects on human brain development and disease^23,27,28^.

The specific objectives of this study are:

i. to explore the genetic architecture of cerebellar lobules by assessing their heritability and identifying patterns of genetic correlation across different cerebellar lobules, modeling potential distinct genetic factors;
ii. to assess whether evolutionary signals that tag different periods in human evolution are enriched or depleted in the heritability of cerebellar substructures, clarifying the influence of evolutionary factors on cerebellar genetics, and
iii. to uncover genetic correlations with other brain regions (both subcortical and cortical) as well as with psychiatric and cognitive traits, thereby elucidating potential genetic links between cerebellar function and mental health.

## Results

In order to gain further insights into possible genetic effects that go beyond the global cerebellar volume, we first performed an in-depth analysis of the genetic architecture of cerebellar substructures. A total of 32 cerebellar volumes were included for analysis, including four global measures (left and right cerebellar cortical volume and cerebellar white matter, from the ‘aseg’ subcortical segmentation^29^), and 28 regional cerebellar measures (from the probabilistic cerebellar atlas^30^): 10 lobules on the right (I-IV, V, VI, Crus I, Crus II, VIIB, VIIIA, VIIIB, IV, X), the homologous 10 additional left lobules and 8 vermis volumes (VI, Crus I, Crus II, VIIB, VIIIA, VIIIB, IX and X) (see Fig. S1). In addition, we also used the data from a recent global cerebellar GWAS^20^. These 33 cerebellar measures consisted of 19 independent variables, estimated with PhenoSpD^31^ using a genetic correlation matrix of all cerebellar measures.

### Genetic architecture of cerebellar lobules

#### Heritability of cerebellar regions

**Figure 1:**
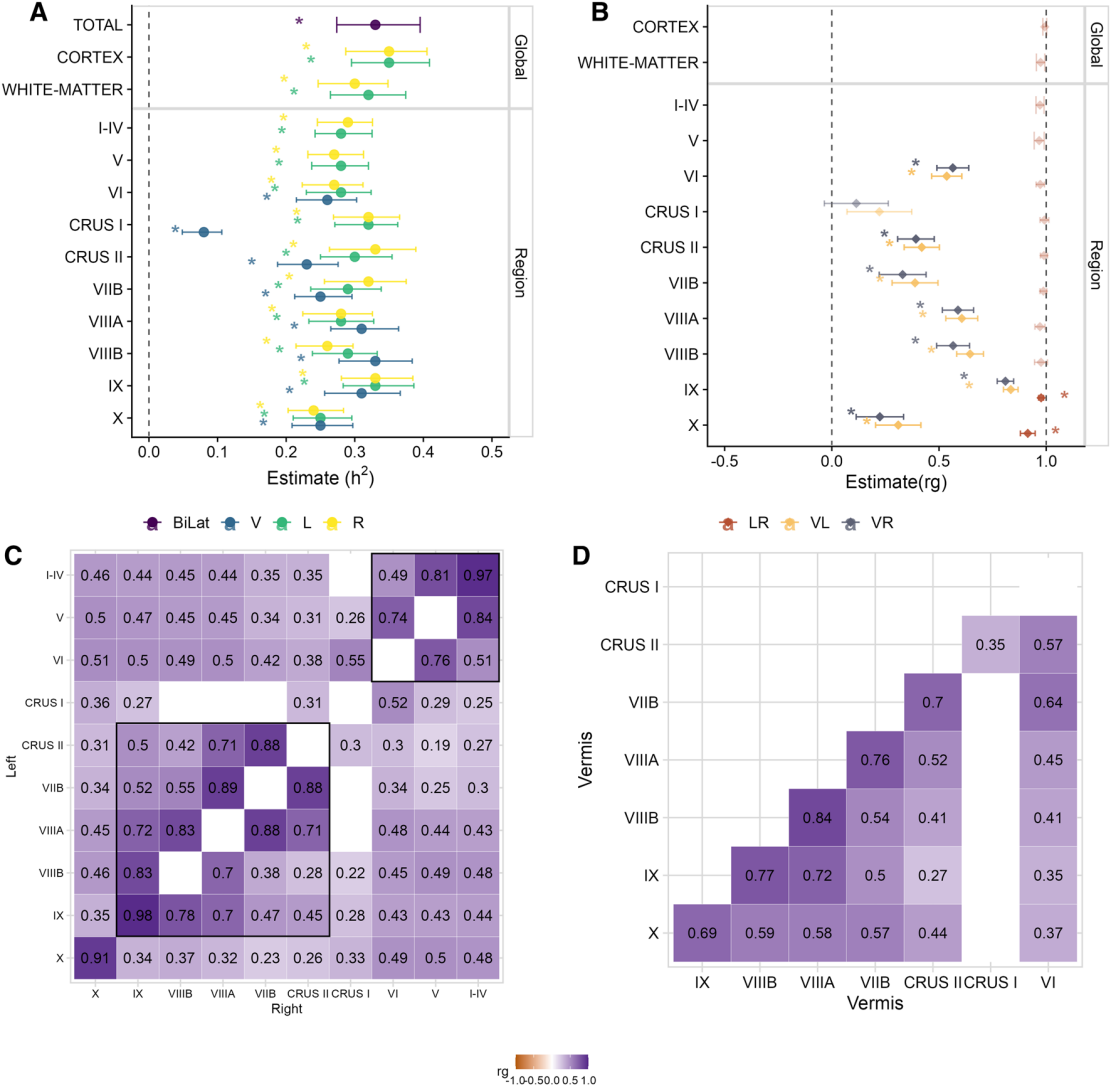
Genetic architecture of cerebellar substructures. (**A**) Heritability of cerebellar volumes. (**B**) Genetic correlations between volumes of a given cerebellar substructure. (**C**) Genetic correlations across cerebellar volumes within each hemisphere. Lower triangle shows the correlation pattern in the right hemisphere; upper triangle shows the correlation pattern in the left hemisphere; the diagonal shows the left-right genetic correlations. The black squares highlight the clustering of lobules based on their genetic correlation. (**D**) Genetic correlations across cerebellar volumes in the vermis. For (**A**) and (**B**) estimates and 95% confidence intervals are shown. For (**C**) and (**D**) empty cells indicate r$_{g}$ estimates that are not significantly different to 0 (triangles) or 1 (diagonal) (after adjustments for multiple comparisons). h$^2$ = heritability; r$_{g}$ = genetic correlation; L = left; R = right; V = vermis; LR = left-right; VL = left-vermis; VR = right-vermis.

We first assessed whether the cerebellar measures were heritable using Linkage Disequilibrium Score Regression (LDSC)^32^. All cerebellar measures were heritable, with estimates ranging from 0.08 for the vermis of Crus I (se = 0.015, adjusted p-value = 2.6e–6) to 0.35 for the left and right cerebellar cortices (se = 0.015, adjusted p-value = 1.2e–32) (all adjusted p-values < 0.05; LDSC intercepts ranging 1.00–1.03, se $\sim$0.01; see Table S2, Fig. 1A). All cerebellar subregions except the vermis of Crus I were also genetically correlated to the mean volume of the cerebellar lobules (range from r$_{g}$ = 0.41 with vermis of Crus II to 0.79 with right lobule VIIIA), as were the global white-matter and cortical cerebellar volumes (see Table S3).

#### Genetic correlations across cerebellar regions

Next, we examined genetic correlations with LDSC within the cerebellum through two complementary analyses. First, we computed the pairwise genetic correlations between vermal, left and right volumes for each lobule (i.e. left-right, vermal-left and vermal-right), and tested whether they were significantly different to 1. Such an imperfect genetic correlation would imply that, despite common genetic factors influencing left, right and vermal volumes for a given lobule, there are also potential genetic factors that would affect each lateral substructure in a specific manner. Left and right volumes were highly correlated for all lobules (r$_{g}>$ 0.9), indicating that most of the genetic effects are shared across cerebellar hemispheres (all but two adjusted p-values > 0.05, alternative hypothesis: r$_{g}$ = 1). Nevertheless, lobules IX and X had a genetic correlation estimate that was significantly lower than 1 (adjusted p-values < 0.05), suggesting that there may be hemisphere-specific genetic effects on these substructures (see Table S3, Fig. 1B). For each lobule, the genetic correlation between the vermis and the lateral (left and right) cerebellar volumes was moderate (median r$_{g}$ = 0.40, see Fig. 1B), with the highest correlation for lobule IX (r$_{g}$(Left-Vermis) = 0.84, se = 0.02), and the lowest for Crus I (r$_{g}$(Right-Vermis) = 0.11, se = 0.08, adjusted p-values > 0.05).

To explore whether there were distinct genetic effects across lobules in the cerebellum, we computed genetic correlations within all cerebellar measures of the left, right and vermal hemispheres separately. This analysis revealed two main clusters that reflect cerebellar anatomy (Fig. 1C): an anterior cluster, encompassing lobules I-IV, V and VI, and a posterior cluster, consisting of lobules Crus II, VIIB, VIIIA, VIIIB and IX. Lobules within each cluster were highly correlated with each other ( e.g. r$_{g}$ left lobule V, left lobule VI: 0.74; r$_{g}$ right lobule V, right lobule VI: 0.76). Crus I and lobule X were moderately correlated with both the anterior (r$_{g}$ range: 0.18 between left Crus I and left I–IV to 0.55 between left Crus I and left lobule VI) and the posterior (r$_{g}$ range: 0.14 between right Crus I and right lobule VIIB to 0.36 between left Crus I and left lobule X) clusters. The correlation pattern for the left and right hemispheres was very similar, while the pattern was a bit different for the vermis (Fig. 1D): lobule X was genetically more correlated with the posterior cluster, while Crus I was only significantly correlated with Crus II (r$_{g}$ = 0.4, se = 0.09, adjusted p-value = 0.0084).

#### Genomic factor analysis

In order to further investigate the genomic clustering patterns within cerebellar substructures we used genomic structural equation modelling (GenomicSEM) to assess the fit of alternative models of shared genetic architecture across cerebellar measures^33^. Given the high genetic correlations between left and right substructures of the cerebellum (all r$_{gL,R}>$0.9, Table S3) we ran two sets of parallel analyses separately, each including 18 cerebellar substructures: either the 8 vermal measures and 10 left-cerebellar measures (Left-Vermis model), or 8 vermal and 10 right-cerebellum measures (Right-Vermis model).

To determine the optimal number of genomic factors that could parsimoniously explain the genetic correlation matrix, we applied four tests (Kaiser, acceleration factor, optimal coordinates and parallel analysis), which indicated that either a one-factor (acceleration factor) or a four-factor model was the best. Next, we fit exploratory factor analyses (EFAs) for four factors to inform confirmatory factor analysis (CFA) models (one and four factors) in a last step. The CFAs were evaluated using model fit indices such as comparative fit index (CFI) and standardized root-mean-squared residual (SRMR) and Akaike Information Criteria (AIC).

The common factor model, in which all cerebellar substructures would share a unique latent global cerebellar factor, did not fit the data well (Left-Vermis model: AIC = 59,020.1, CFI = 0.81, SRMR = 0.11; Right-Vermis model: AIC = 172,958, CFI = 0.67, SRMR = 0.12), suggesting that it is likely to be misspecified (Tables S4, S5).

**Figure 2:**
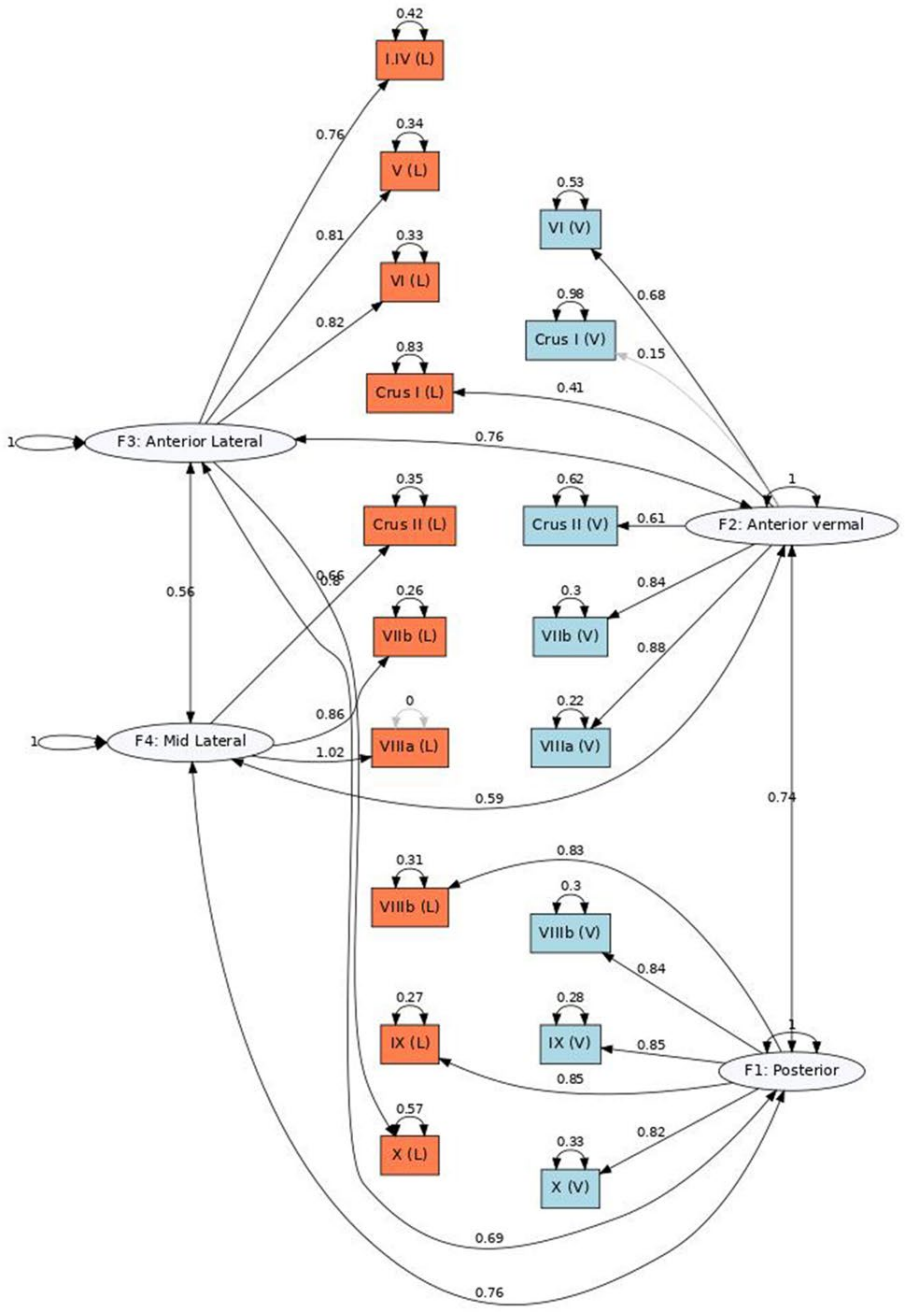
Path diagram of the genomic SEM CFA for the Vermis-Left four-factor correlated model. The genetic components are represented as white circles, to indicate latent factors (i.e. not directly observed). Left-cerebellar measures are indicated by orange squares, vermal-cerebellar structures are indicated by light-blue squares. Non-significant paths are indicated in grey. All parameter estimates are standardized. Model fit indices and unstandardized results are available in Tables S4 and S7. V = vermis; L = Left.

Next we fit exploratory factor analysis with four factors. On the four factor CFA each cerebellar substructure was assigned to a factor when their standardized loading in the EFA was $>0.5$ (Table S6). In the cases where a given substructure did not achieve a loading of 0.5, this substructure was assigned to the factor with the largest standardized loading. These four factor CFAs fit the data well ( Left-Vermis model: AIC = 23,487.3, CFI = 0.93, SRMR = 0.08; Right-Vermis model: AIC = 53,378.27 , CFI = 0.90, SRMR = 0.08, see Table S4). The factor structure revealed from both Left-Vermis and Right-Vermis models was almost identical (Table S7), the Left-Vermis model is illustrated in Fig. 2. This includes factors that could be approximately described as representing an anterior lateral factor (lateral I-IV, V, VI, X), a mid-lateral factor (lateral Crus II, VIIb, VIIIa), an anterior vermal factor(lateral Crus I, and vermal VI, Crus I, Crus II, VIIb, VIIIa ) and a posterior (lateral and vermal VIIIb and IX, and vermal X) factor. The four factors correlated with each other (standardized estimates > 0.45). For most of these measures, there is still significant residual variance that is not accounted for by these factors, suggesting additional genetic heterogeneity. This analysis further supports that the cerebellum is not a genetically homogeneous structure, and shows evidence for differential genetic effects across the anterior-posterior and vermal-lateral axes.

### Stratified heritability and evolutionary considerations

We also examined whether specific evolutionary genomic annotations (i.e. datasets consisting of genomic regions of evolutionary relevance) are depleted or enriched in heritability across the 33 cerebellar measures. We used stratified LDSC (S-LDSC)^34^ to compute the contribution of variants within a given genomic annotation towards trait variation, and assess whether this contribution is larger or smaller than would be expected given the relative proportion of variants in that region. We considered six human-gained genetic and epigenetic sequence elements as genomic annotations marking different evolutionary periods (similar to the approach in^35^): adult and fetal Human Gained Enhancers^36^, Human Accelerated Regions^37^, Ancient Selective Sweeps^38^, SNPs introgressed from other hominins^39^ and genomic regions depleted of such introgression signals (so-called “introgression deserts”)^40^.

**Figure 3:**
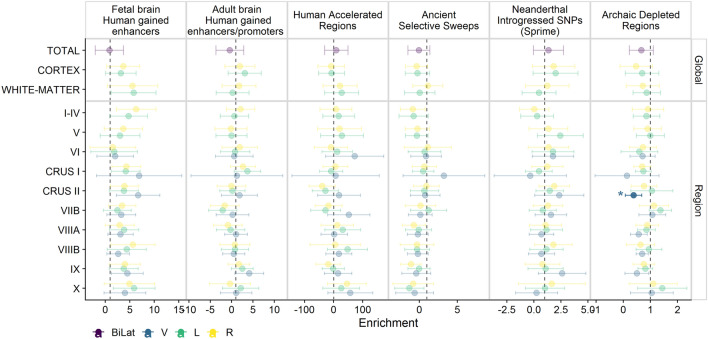
Stratified heritability analysis for six human-gained genetic and epigenetic sequence elements as genomic annotations marking different evolutionary periods. Each point reflects the enrichment estimate and error bars indicate the 95% confidence intervals. Estimates with a Bonferroni adjusted p-value < 0.05 are highlighted with a brighter colour and marked with an ﻿‘*’. Bilat = bilateral measure; V = Vermis; L = left; R = right.

Only the vermis of Crus II showed a significant heritability depletion for so-called large introgression deserts (h$^2$(C) = 0.38, s.e. = 0.15, adjusted p-value = 0.0268, see Fig. 3)^40^. No other cerebellar measure showed a significant enrichment or depletion in any of the annotations (Table S8).

### Global genetic correlations with other subcortical and cortical brain measures, psychiatric disorders and cognitive traits

#### Subcortical volumes

**Figure 4:**
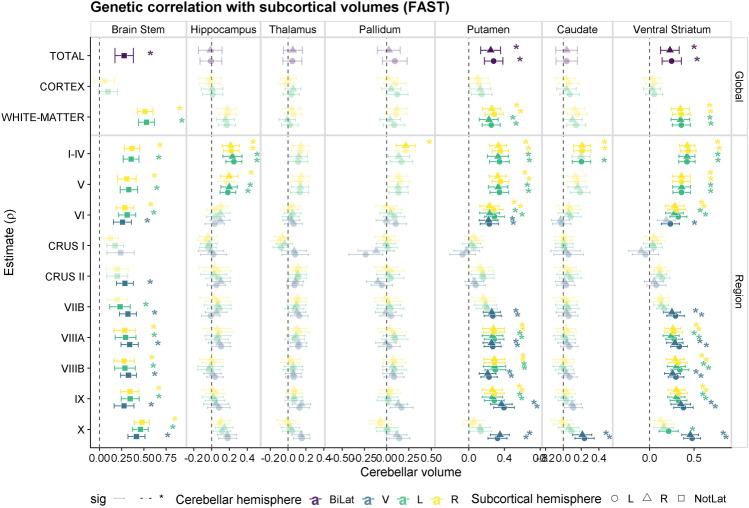
Genetic correlations between cerebellar measures and subcortical volumes (‘FAST’﻿ parcellation). Each point reflects the genetic correlation estimate and error bars indicate the 95% confidence intervals. Estimates with a Bonferroni adjusted p-value < 0.05 are highlighted with a brighter colour and marked with an ﻿‘*’. Bilat = bilateral ; V = Vermis; L = left; R = right; NotLat = not lateralized.

We found several genetic correlations between the cerebellar substructures and other subcortical volumes as defined by the Harvard-Oxford subcortical atlas (Fig. 4, Table S9). The brainstem, putamen and ventral striatum had positive correlations with most global cerebellar measures, except for cerebellar cortex volumes. Similarly, the regional cerebellar volumes had genetic correlations with the brainstem, putamen and ventral striatum, except for a few measures (i.e. Crus I and Crus II), and a lower genetic correlation with lateral VIIB and X lobules. We did not find any genetic correlations between the thalamus and any cerebellar measures. The pallidum was genetically correlated with the right cerebellar lobule I-IV, but not with the global measures. Overall, the genetic correlation patterns were quite stable across both hemispheres and the vermis, although there are also some noteworthy observations: bilateral lobule X measures were correlated with the brainstem but no other subcortical structures, while the vermis of lobule X was positively correlated with the putamen, caudate and ventral striatum.

To assess the robustness of these signals, and to enable a direct comparison with previous studies^20^, we also computed the genetic correlations with subcortical volumes from the ‘aseg’ segmentation^29^ (see Fig. S5, Table S9): a similar trend can be observed for the brainstem, and also showed genetic correlations with the pallidum (most of the global measures except the cerebellar cortex and all the regional measures except for Crus I and Crus II). On the other hand, significant correlations with the thalamus were revealed by this sensitivity analysis (cerebellar lobules I-IV, V and X and white-matter cerebellar volume; r$_{g}$ range: 0.19–0.31, all Bonferroni adjusted p-values<0.05), while the r$_{g}$’s were weaker with the putamen or accumbens (as reported by^20^ for the global cerebellar volume).

#### Cortical volumes

**Figure 5:**
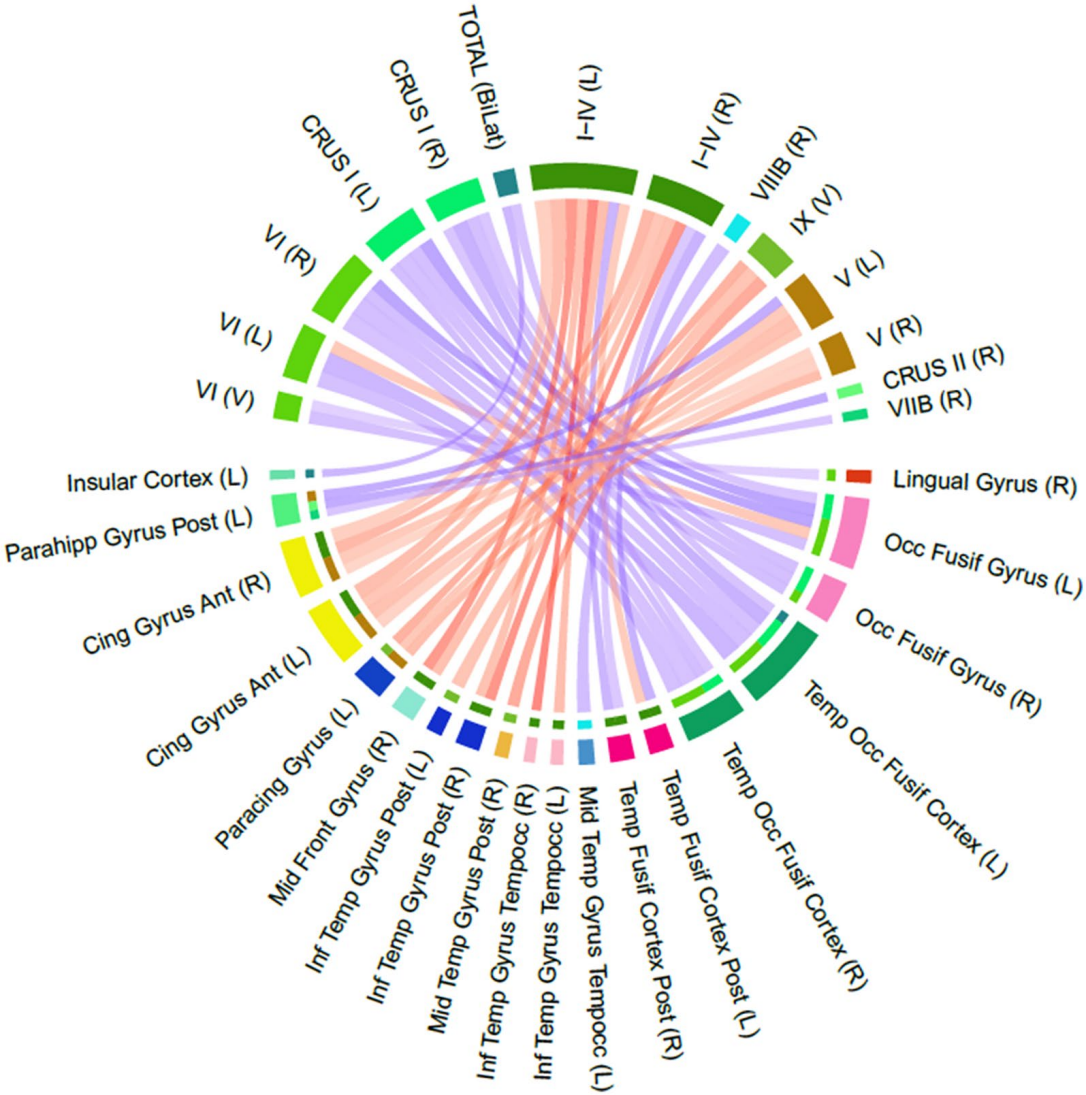
Genetic correlations between cerebellar measures (upper half) and cortical volumes (Harvard-Oxford atlas ‘FAST‘ parcellation; lower half). Each significant genetic correlation is depicted as a connection between any cerebellar (upper half) and cortical (lower half) regions. The strength of the genetic correlation is reflected by the shade of the connection between two regions (blue = positive and red = negative). For each of the cortical regions, the colors indicate the cerebellar origin for each connection. Only cortical regions with at least one significant genetic correlation with cerebellar lobules are represented in this figure. All genetic correlation estimates are available in Table S10. Bilat = bilateral; V = Vermis; L = left; R = right; NotLat = not lateralized.

In order to gain insights about the shared genetic influences of the different cerebellar lobules in cortical regions, we assessed genetic correlations between cerebellar volumes and 96 cortical volumes (48 for each hemisphere) as defined by the Harvard-Oxford atlas^41^. There were 46 cerebellar-cortical correlations that were significantly different from zero after adjusting for multiple comparisons (Fig. 5, Table S10). Total cerebellar volume was significantly correlated with two cortical measures: positively (r$_{g}$ = 0.27, se = 0.06) with the left temporal occipital fusiform cortex and negatively with the left insular cortex (r$_{g}$ = − 0.20, se = 0.05).

The rest (44/46) of the significant cortico-cerebellar genetic correlations were with specific cerebellar lobules. Over 80% (17/20) of the positive correlations were between cerebellar lobules VI and Crus I and occipito-temporal cortical regions: left and right cerebellar measures correlated with the temporal occipital fusiform and occipital fusiform gyrus ipsi- and contra-laterally (r$_{g}$ range: 0.24,0.49), while vermal lobule VI had a r$_{g}$ = 0.28 with right lingual gyrus and r$_{g}$ = 0.31 with left occipital fusiform gyrus. Right Crus II, right lobule VIIB and left lobule V had positive r$_{g}$’s with posterior parahippocampal gyrus in the left hemisphere (r$_{g}$ range: 0.21, 0.26).

Most negative correlations were driven by lateral anterior cerebellar lobules (lobules I–IV and V) with the paracingulate gyrus and the anterior cingulate gyrus (r$_{g}$ range: − 0.23, − 0.38). In addition, lobule I-VI was negatively correlated (r$_{g}$ range: − 0.25, − 0.35) with the right middle frontal gyrus and inferior temporal cortical regions (posterior inferior temporal gyrus, temporoccipital ITG and temporal occipital fusiform cortex), while vermal IX also had a negative genetic correlations with the paracingulate gyrus (left), and right MTG and left ITG (r$_{g}$ range: − 0.25, − 0.36). The right VIIIB had a negative r$_{g}$ of − 0.35 with the left temporo-occipital MTG.

#### Psychiatric disorders and cognitive traits

In the same vein, we assessed global genetic correlations with ASD, SCZ and cognitive performance (measured as fluid intelligence, see methods section). These analyses yielded relatively low genetic correlations (maximum absloute $r_{g}$ of 0.2) that were not significant after multiple testing correction (all adjusted p-values > 0.05, see Fig. S3 and Table S11).

## Discussion

Overall, our results confirm that the cerebellum is a heritable structure, both globally and at the regional level, and highlight the variability across its different anatomical subdivisions. They therefore support further investigation of the relationship between specific cerebellar substructures and other traits.

The SNP-h$^{2}$ estimates for most cerebellar substructures were moderate (> 0.2 for 27/28 measures), ranging from 0.23 (95% CI = 0.19-0.28) for vermis of Crus II, and a maximum estimate of 0.33 for left and right lobule IX (95%CI = 0.28–0.39). The vermis of Crus I showed the lowest heritability (0.08, 95% CI = 0.05–0.11), albeit significantly different from zero. Of note, the vermis of Crus I, designated according the cerebellar probabilistic atlas^30^, corresponds to the vermal component of VIIAf and has a markedly smaller volume (mean volume = 2 mm$^{3}$) compared to other lobules (mean volumes ranging from 139 mm$^{3}$ for vermal VIIB to 11,432 mm$^{3}$ for right Crus II). This discrepancy in volume may contribute to the lower heritability estimate observed in this region, potentially due to increased measurement error associated with smaller volumes. This analysis confirms that most of the regional cerebellar volumes are partially influenced by genetic factors^20,23^, to a similar extent as cortical^23,42^ and subcortical volumes^23,43^. While the heritability estimates had a similar magnitude (95% confidence intervals overlapped for all except vermis of Crus I), the specific genetic influences could vary between the hemispheres and across the regions.

Thus, we next assessed the genetic correlation between the left, right and vermal measures for a given cerebellar substructure. The vermal measures only had a low to moderate genetic correlation with corresponding left and right hemispheric measures, while genetic correlations between most left and right measures were significantly not different to one. Only left and right of lobules XI and X had a genetic correlation that was significantly different albeit very close (rg > 0.9) to one, implying potential specific genetic effects on these posterior lobules. In fact, the largest difference was observed for lobule X, which constitutes the flocculonodular lobe, the phylogenetically oldest part of the cerebellum. In principle, we could have expected lower genetic correlation between hemispheres also for other more anterior lobules that show some asymmetrical activation during motor control (I-V) or cognitive tasks such as language (VI, Crus I)^44,45^. However, existing evidence does not support a direct link between structural cerebellar asymmetries and handedness^46^. Functional lateralization does not necessarily imply anatomical asymmetries, and even when present, anatomical asymmetries are not entirely of genetic origin^47,48^. Recent studies have shown that the largest cortical brain asymmetries have only a moderate SNP-based heritability (SNP-h2 < 0.15)^47,48^, while estimates for handedness, which has a moderate genetic component in twin studies, are much lower (SNP-h2 range 0.03–0.10)^49,50^. In summary, our findings suggest that genetic influences are largely shared between the left and right cerebellar substructures, while these influences differ somewhat for the vermis.

When considering the left, right and vermis measures separately, almost all within hemisphere genetic correlations were significantly different to zero, with moderate to high positive correlations, reflecting a shared cerebellar genetic component. Nevertheless, we also observed some clustering that mirrored the anatomical division of the anterior (lobules I-IV, V, VI) and posterior cerebellar lobes (Crus II, VIIA, VIIB,IX), which has been claimed to distinguish between the motor and affective/cognitive cerebellum^12^. This pattern was identical in the left and right cerebellar hemispheres. Through genomic SEM analyses, we confirmed that a model with four genetic factors explained these correlation patterns well. These factors could be roughly expressed as an anterior lateral, mid-lateral, anterior-vermal and posterior. Again, these results highlight that there is genetic heterogeneity within cerebellar substructures.

In order to shed light on the specific evolutionary scales that could have disproportionately influenced the heritability of the cerebellar measures, we performed an enrichment analyses for evolutionary annotations, which are intended to capture different timeframes in human evolution: from adult and fetal Human Gained Enhancers^36^ (> 30 million years ago, since human’s common ancestor with Old World Monkeys), to genomic regions that have undergone rapid change in the human lineage (Human Accelerated Regions) since the last common ancestor with great apes, about 7 million years ago^37^, to more recent Ancient Selective Sweeps^38^ ($\sim$ 250,000 years ago), and SNPs introgressed from other hominins^39^ and genomic regions depleted of such introgression signals (so-called “introgression deserts”)^40^. Given the rapid expansion of the cerebellum in the hominin lineage relative to other brain regions^16,51^, we expected that variability within genomic regions that have undergone changes in this lineage would also disproportionately influence heritability in (some) cerebellar regions. A depletion of heritability was detected for Crus II Vermis in the context of “deserts of introgression”^40^. These regions of the genome are depleted of introgressed archaic haplotypes and enriched for genes expressed in the human brain, specifically in regions such as the cerebellum, the striatum and the thalamus^18,52^. The pattern observed here could be interpreted as the consequence of strong purifying selection against introgressed archaic variants, leading to a fixation of the modern variants in these “deserts of introgression” and hence a lower proportion of variance being explained by genetic variation within such regions, as has been suggested to explain the heritability depletion of cortical surface area measures in archaic deserts^53^. This explanation remains speculative but, in support of this idea, the vermis of Crus II also showed a positive enrichment of fetal brain human gained enhancers, although this did not survive multiple testing correction and multiple other cerebellar regions also show the same trend. The lack of enrichment of heritability in all other evolutionary annotations for the rest of cerebellar measures is in line with another study which, using an alternative approach, failed to find an enrichment of total cerebellar volume GWAS signal in human accelerated regions^21^.

The cerebellum has many connections with other cortical and subcortical regions. We aimed to understand whether these relationships may be driven by a shared genetic component. Global genetic correlations between total cerebellar volume and subcortical structures confirmed some of the previously reported genetic correlations^20^, while our region-specific analysis provides greater resolution regarding the potential source of global cerebellar signals. There was a robust genetic correlation between cerebellar volumes and the brainstem possibly reflecting that the brainstem consists of the major cerebellar input^54^. This result was consistent across analyses (i.e. using different parcellations for the cerebellar structures), and replicated previous studies that used different GWAS summary statistics for the subcortical structures^20^. We did not find any significant genetic correlation between the thalamus and total cerebellar volume in either of the parcellations we used, in contrast to the previously reported genetic correlation of 0.24 between the thalamus (average left and right) and global cerebellar volume^20,43^. There was, however, significant genetic correlation with some cerebellar lobules (I–IV, V and X) using the ‘aseg’ parcellation for the thalamus volume. Further, we also confirmed genetic correlations between the cerebellum and the striatum: although there was variability with regard to the subcortical parcellation used for the striatal volume (putamen in Harvard-Oxford vs pallidum in ‘aseg‘) and the specific cerebellar measures (not with Crus I and Crus II). Analyzing specific lobules also revealed new genetic correlations between cerebellar measures and subcortical structures, including general positive genetic correlations between cerebellar lobules (except Crus I, Crus II and lateral X) and and ventral striatum/accumbens. This highlights that the genetic correlations that are currently being detected in genetic studies are sensitive to the specific datasets and/or the analysis parameters used (such as the parcellation). The lack of genetic correlations of Crus I/II with subcortical structures could be potentially linked to their more recent evolutionary trajectory. There were also some genetic correlations restricted to specific cerebellar lobes: such as lobules I–IV and V with the hippocampus, or lobules I–IV and vermal X with the caudate. Of note, we only included global volumes of other subcortical structures in these analyses, and assessed whether these have differential genetic correlations with the cerebellar substructures. In order to gain further resolution of these genetic relationships, future work could consider regional measures within the other subcortical structures as well, such as the thalamic nuclei^55^ or hippocampal subfields^56^.

We next considered cerebello-cortical genetic correlations, which could give us information about shared implications for cognitive processes. This analysis revealed two main genetic correlation patterns: on the one hand, a cluster of negative genetic correlations between the most anterior cerebellar lobules (lateral I–IV and V) with anterior cingulate and paracingulate gyri; on the other hand, a cluster of positive genetic correlations between cerebellar lobules VI and Crus I with occipito-temporal cortical regions including the fusiform. These effects were not lateralized, since similar genetic correlations were observed both ipsi- and contralaterally, with the strongest r$_{g}$ being between lobule VI and temporo-occipital fusiform cortex. A recent study assessing cerebro-cortical covariance patterns also highlighted the presence of an ipsilateral axis of cortical-cerebellar variation^57^ . Although the cortical areas with the strongest genetic correlations are also the regions just superior and contiguous with the cerebellum as a whole, it should be noted that the effects are only present for specific cerebellar lobules, which indicates some specificity that would not be expected if the effects were due to some estimation bias or technical challenges. The identified genetic associations highlight cortical regions and cerebellar lobules with functional roles in language processing, visual processing and cognitive control. For instance, Crus I, lobule VI and lobule IX have been previously implicated in language processing within the cerebellum through a fMRI language contrast (“story > math”)^58^, while the fusiform cortex is known to be involved in visual processing, the left fusiform being involved in the orthographic mapping in reading, including word recognition. Interestingly, a meta-analysis reported reduced grey matter volume in bilateral lobules VI and right Crus II in dyslexia^59^, while bilateral activation in lobule VI and Crus I has been linked to orthographic processing, with dyslexic children showing stronger functional connectivity between right cerebellar lobule VI and left fusiform gyrus during an orthographic task^60^, although others report a lack of functional connectivity to the mid fusiform gyrus in the cerebellum^61^. On the other hand, anterior cerebellar lobules (lateral I–IV and V) were genetically negatively correlated with volumes in the cingulate cerebral cortex, which is interesting given the cerebellum’s function in error processing and cognitive control, although more posterior lobules have been typically implicated^10,62^. It is important to stress that the nature of the present study is purely correlational, examining genetic associations between brain structural volumes. Furthermore, the effect we observed is not lateralized (as many cognitive functions are), and it therefore has no hemispheric specificity. Nevertheless, we hypothesize that shared genetic contributions to these specific cerebellar and cortical regions could impact their ability to optimally host processes in which both are involved, such as complex visual processing or error monitoring.

In line with previous studies^20,21^, cerebellar volumes did not show whole-genome level correlation with ASD, SCZ or cognitive performance. Our power analysis indicates that we were well-powered to detect moderate to high genetic correlations of cerebellar lobules and these traits (see Fig. S4). We take the lack of genetic correlations to indicate that there are no such shared genetic effects that are consistently shared across the genome. This could imply that despite phenotypic correlations between these psychiatric disorders and cerebellar measures^14,63,64^, the associations do not arise from a shared genetic architecture. Of note, we only considered global (whole-genome) genetic correlations, with risk of cancelling out potential negative and positive genetic correlations across the genome. More nuanced analyses such as local genetic correlations^65,66^ or methods that assess cross-trait enrichment in the GWAS signal (e.g. conditional-FDR^67,68^) could also be considered in the future. These approaches allow for positive and negative genetic correlations for different genomic loci, and are therefore more sensitive than global genetic correlations. For instance, a study^21^ identified some significant local genetic correlations between total cerebellar volume and SCZ, Alzheimer’s disease and Parkison’s disease, in spite of lack of global genetic correlations, and another study^20^ reported an enrichment of SCZ, bipolar disorder and ASD associated signals within total cerebellar GWAS results. However, conducting such an analysis was deemed beyond the scope of the present study for two main reasons. First, considering the extensive dataset comprising 33 cerebellar phenotypes and thousands of genomic loci, conducting local genetic correlation analyses would entail a significant increase in multiple comparisons. This would not only decrease statistical power but also complicate the interpretability of the results. Second, the existing literature indicates only moderate consistency across methods used to assess local bivariate genetic correlations^65,66^. This inconsistency further discouraged us from pursuing such an analysis across multiple phenotypes in this study. Therefore, we suggest that future research with a more hypothesis-driven targeted approach may be better suited to explore this avenue. Such an approach could provide a more comprehensive understanding of the relationship between colocalizing genomic signals.

Two caveats should be noted regarding the present study. First, it relied on publicly available GWAS summary statistics from the UK Biobank dataset for all the neuroimaging traits analyzed. Although this is a powerful resource and one of the largest and most homogeneous brain imaging genetics datasets to date^23^, the participants in this dataset are of relatively advanced age (mean age at recruitment over 56, see Table S12) and participation bias in the UK Biobank is known to distort the genetic correlation estimates^69,70^. Furthermore, we leveraged summary statistics that were readily available from previous studies^23^, which restricted our ability to assess the sensitivity of the results (e.g. by including or excluding covariates to identify potential confounds or collider effects). The results presented in this study should therefore be replicated in studies using different designs that would enable their generalizabilty to be tested. Second, our study focused on volumetric measures of cerebellar lobules, which may not fully capture the functional organization of the cerebellum^7,71^. A task-based functional parcellation of the cerebellum has provided a detailed mapping of its functional organization, and highlighted its involvement in cognition^7^, and this work has been further validated by extending it to multiple datasets to create a hierarchical atlas of the human cerebellum^71^. While functional parcellation offers a more nuanced understanding of cerebellar function, structural and functional parcellations are highly correlated, for instance when assessing structural cerebelo-cortical covariation^57^. This suggests that anatomical parcellation, even though not perfect, can still provide meaningful insights into cerebellar organization. While recent advancements in functional parcellation have provided detailed mappings of cerebellar function, our study relied on anatomical parcellation due to its widespread use and availability. Using functionally defined regions in future studies will be essential to gain a more comprehensive understanding of the genetic relationships between cerebellar phenotypes and other brain, psychiatric, or cognitive phenotypes.

In summary, we have performed a comprehensive genetic analysis of cerebellar volumes, which has provided new insights regarding the genetic relationships that are shared or unique between specific cerebellar lobules and other brain regions. It is also noteworthy that our study failed to detect strong lateralization effects, possibly pointing to the relevance of both hemispheres even in domains (such as speech) where lateralization effects are expected^72^.

## Methods

### Data

#### GWAS summary statistics

GWAS summary statistics for structural (T1) imaging derived phenotypes (IDPs) based on the UK Biobank (N $\sim$ 31,000) were downloaded from the Oxford Brain Imaging Genetics Server-BIG40 (https://open.win.ox.ac.uk/ukbiobank/big40/)^23^. Table S12 contains descriptive statistics of the UK Biobank resource, including age, sex, ICD10 codes for relevant mental and nervous system disorders, addiction prevalence and operative procedures.

We refer the reader to the original publication on genome-wide association studies of brain imaging phenotypes in UK Biobank^23^ for details about how the imaging quality control and GWASes were performed. We note briefly, that these GWAS were run on brain imaging data was from the 40,000 participant release from early 2020, which was processed by WIN/FMRIB on behalf of UK Biobank as described in^73^. Given the vast amount of subjects and images, this pipeline includes an semi-automatic quality control (QC) tool to identify problems either in the acquisition or in later processing steps. For this, a trained automated classifier scores all datasets for quality (including a classification of problems and imperfections for the T1 that encompass data incompleteness and “structurally atypical”), and any T1 that is close to the “bad data” threshold is carefully manually reviewed to ensure data quality^73^. Next, the T1 pipeline includes a gradient distortion correction, cuts down the field of view, calculates a registration to the standard atlas (MNI152), applies brain extraction, performs decaying and segments the brain into different tissues and subcortical structures^73^. For the GWAS analysis, IDPs were deconfounded for a set of potential imaging confounds (including confounds for age, head size, sex, head motion, scanner table position, imaging center and scan date-related slow drifts), as well as the 40 population genetic principal components^23,74^). The GWAS summary statistics used in the current study come from the subset of $\sim$ 33,000 unrelated samples with recent UK ancestry and accepted genotyping and imaging quality control (discovery and replication datasets combined)^23^.

The selected IDPs for the current study included a total of 33 cerebellar measures: 10 volumes of cerebellar regions (28 measures in total: left, right and vermis for 8 measures, left and right only for 2 measures; Harvard-Oxford subcortical parcellation), plus two global measures: cerebellar cortical volume and cerebellar white-matter volume (left and right)^29^. In addition, we also included the summary statistics from the recently published total cerebellar volume GWAS, in which total cerebellar volume had been computed as the sum of all the aforementioned FAST cerebellar volumes except for the Crus I vermis volume^20^. The inclusion of this global trait served as a control to ensure whether lobule specific effects were not mirroring this global signal. Additional brain volumes were also used for some analyses (see Table S1 for all measures). These included subcortical volumes (13 subcortical volumes from the Harvard-Oxford atlas; 15 from the subcortical atlas ‘aseg’^29^), and the 96 cortical volumes (Harvard-Oxford atlas^41^). All GWAS summary statistics used in the current study are specified in Table S1.

#### Genetic quality control procedures

For each GWAS summary statistic dataset we applied standard quality control filters to keep unique unambiguous SNPs present in HapMap3 that had minor allele frequency (MAF) > 1%. All datasets adopted hg19 genomic coordinates. The LD scores used for LDSC were calculated using the European subsample of the 1000G phase3 project (https://data.broadinstitute.org/alkesgroup/LDSCORE/), excluding the major histocompatibility complex (MHC) region.

### Estimating number of independent cerebellar traits

PhenoSpD was used to define the number of independent traits across these 33 cerebellar measures^31,75^. PhenoSpD uses GWAS summary statistics to first estimate the phenotypic correlations across traits, and then applies spectral decomposition of matrices to identify the number of independent variables^31,75^. We used the estimated effective number of independent variables (‘VeffLi’) to adjust using Bonferroni correction for multiple comparisons across the independent cerebellar measures (see Fig. S1).

### SNP-based heritability

SNP heritability ($h^{2}_{SNP}$) is the proportion of variance explained by common genetic factors, and was computed using GWAS summary statistics by running the Linkage Disequilibrium Score regression (LDSC, v1.0.1)^32^. LDSC allows the computation of $h^{2}_{SNP}$ and genetic correlation (see below) from GWAS summary statistics, without relying on individual level data. This method leverages the relationship between test statistics and linkage disequilibrium (LD) to disentangle true polygenic signals from confounds, such as population stratification or cryptic relatedness. Specifically, LDSC performs a regression of the test-statistic from the GWAS on the LD scores, providing a measure of confounding effects (intercept), where deviation from 1 can be interpreted as an index of stratification/confounding, and the slope is an estimate of SNP heritabilty, i.e. how much the test-statistic tracks with changes in LD^32^.

The significance of the heritability estimates was Bonferroni adjusted for multiple comparisons for the 19 effective independent cerebellar traits as defined by PhenoSpD (see above; p-value threshold = 0.05/19 = 0.0026).

We computed a Pearson’s correlation coefficient between mean heritability estimates and the mean volume of each cerebellar lobule. The descriptive statistics (mean and standard deviation) of each measure were extracted from the UK biobank’s showcase (https://biobank.ndph.ox.ac.uk/showcase/) searching for their data field ID (UKB_ID in Table S1). The descriptive statistics from “Instance 2: Imaging visit (2014+)” were used for this analysis (N = 42,798) (Fig. S2).

### Global genetic correlations

The genetic correlation (r$_{g}$) between two traits is the proportion of shared variance explained by common genetic factors. Bivariate LD Score regression is a robust estimator of genetic correlation that can be derived from GWAS summary statistics^76,77^. LDSC (v1.0.1) was used to estimate global genetic correlations between pairs of traits.

For every pair of measures for the left and right volumes of each cerebellar structure, we tested whether r$_{g}$ was significantly different to 1, whereas for analyses involving different cerebellar measures or non-cerebellar measures, we tested whether the genetic correlation estimate was significantly higher than 0.

Genetic correlations within the cerebellum were computed for a given cerebellar volume between left-right, left-vermis and right-vermis volumes; for the different volumes within the left-hemisphere, right-hemisphere and vermis (200 r$_{g}$ in total, p-value threshold = 0.05/200 = 0.00025).

Cerebellar volumes were also tested for genetic correlations with subcortical measures, cortical volumes, ASD, SCZ and cognitive performance. Multiple comparison corrections were applied using the Bonferroni method: correcting for 19 effective independent cerebellar traits and the number of other traits within each analysis, which were: 3 cognitive/disorder traits(p-value threshold = $0.05/(19\times 3) = 0.00088$); 13 (‘Harvard-Oxford’) or 15 (‘aseg’) subcortical volumes (p-value threshold = $0.05/(19\times 13) = 0.0002$; p-value threshold = $0.05/(19\times 15) = 0.00017$); 96 cortical volumes (p-value threshold = $0.05/(19\times 96) = 2.7e{-}05$).

We estimated the minimum power to detect genetic correlations across the cerebellar measures and cognitive/disorder traits at two levels of alpha (0.05 and 0.00088) using the GCTA-GREML power calculator^78^. Tables S1 and S2 summarize all the parameters per trait that went into the power calculations (heritability estimates from LDSC, sample sizes). Since LD score regression^32^ utilize summary statistics while GCTA relies on the individual genotype data, the true power is likely to be slightly lower for LDSC; however, the GCTA-GREML power calculator gives an indicative estimate.

### Genomic structural equation modelling

To further disentangle the genetic correlations across cerebellar substructures, we used GenomicSEM (version 0.0.5)^33^. To this aim, we used the LDSC formatted (munged sumstats) for the 28 cerebellar substructures: 10 left, 10 right and 8 vermal, as specified in Fig. S1, to fit two separate genetic correlation matrices, each with 18 cerebellar measures: 10 lateral (either left or right) and 8 vermal. We did not include the most complex matrix with all 28 cerebellar substructures, as left and right cerebellar measures all had a high genetic correlation of $>0.9$.

For each model, we first generated the genetic correlation matrix, and applied the Kaiser, parallel analysis, acceleration factor and optimal coordinates rules to it in order to determine the number of genomic factors that could be used to parsimoniously represent the data. These results pointed towards either a four-factor (Kaiser, optimal coordinates, parallel analysis) or single common factor (according to acceleration factor). Next, exploratory factor analyses (EFAs) with the *factanal* R package. Finally, confirmatory factor analyses (CFAs) were run using Genomic SEM. Two CFA models were fit for the Vermis-Left and Vermis-Right separately: a common-factor model and a four-factor correlated factors model. The four-factor CFA was specified based on the loadings from the EFA: each measure was assigned to a factor when their standardized loading in the EFA was $>0.5$. In the cases where a given substructure did not achieve a loading of 0.5, this measure was assigned to the factor with the largest standardized loading. These models were evaluated using the following model fit metrics, acceptable model fit being indicated by comparative fit index (CFI) > 0.90 and standardized root-mean-squared residual (SRMR) < 0.10. Akaike Information Criteria (AIC) was also considered, with lower values indicating better fit (balancing overall model fit with number of estimated parameters)^79^.

### Stratified heritability analysis

We used stratified LDSC (S-LDSC)^34^ to compute the contribution of variants within each specific genomic region towards trait variation, and assess whether this contribution was larger or smaller than would be expected given the relative proportion of variants in that region. We considered six human-gained genetic and epigenetic sequence elements as genomic annotations marking different evolutionary periods (similar to the approach taken in^35,80^): fetal brain Human Gained Enhancers (HGE)^36^, adult HGE and promoters in the cerebellum^81^, Human Accelerated Regions^37^, Ancient Selective Sweeps^38^, SNPs introgressed from other hominins^39^ and genomic regions depleted from such introgression signals (so-called “introgression deserts”)^40^.

For each of these evolutionary categories, annotations and LD-scores were created following instructions from the LDSC wiki (https://github.com/bulik/ldsc/wiki/LD-Score-Estimation-Tutorial). We then estimated heritability enrichment or depletion for each category, using the baselineLDv2.2 model (which includes 97 annotations including several other regulatory elements, linkage statistics and measures of selective constraint). For human gained enhancers and promoters (i.e. fetal HGE and adult human gained enhancers and promoters), epigenetic marks from the fetal brain (E081 and E082) and adult brain (E073) from the Epigenome Roadmap Project 25 state model were also included in the model^82^.

## Supplementary Information

Supplementary Information.

Supplementary Tables.

## Data Availability

GWAS summary statistics used in this study (listed in Table S1) are available from the NHGRI-EBI GWAS Catalog and from Oxford Brain Imaging Genetics Server - BIG40.

## Code availability

The custom code associated with this study is publicly available at https://github.com/amaiacc/MS-cerebellum-UKB.
