# Supplementary material for: Insights into the genetic architecture of cerebellar lobules derived from the UK Biobank
Source: Sci Rep. 2024 Apr 25;14:9488. doi: 10.1038/s41598-024-59699-9 (PMC11551202; doi:10.1038/s41598-024-59699-9)
Supplement: Supplementary file 1 — Supplementary Information. [file 41598_2024_59699_MOESM1_ESM.pdf]

Supplementary information

Supplementary tables

"Supplementary Dataset" contains the following supplementary tables:

Table S1. Publicly available genome wide association study (GWAS) summary statistics used in the current study.

Table S2. Heritability estimates for 33 cerebellar measures.

Table S3. Genetic correlation estimates within the cerebellum.

Table S4. Cerebellar genomic SEM model fit indices.

Table S5. Cerebellar genomic SEM common factor model results.

Table S6. Exploratory factor analysis loadings per factor.

Table S7. Cerebellar Genomic SEM confirmatory factor analysis results.

Table S8. Heritability enrichment results for evolutionary annotations.

Table S9. Genetic correlation estimates between cerebellar and subcortical volumes.

Table S10. Genetic correlation estimates between cerebellar and cortical volumes.

Table S11. Genetic correlation estimates between cerebellar volumes and psychiatric disorders and cognitive traits.

Table S12. Demographic information about the UK Biobank.

Supplementary figures

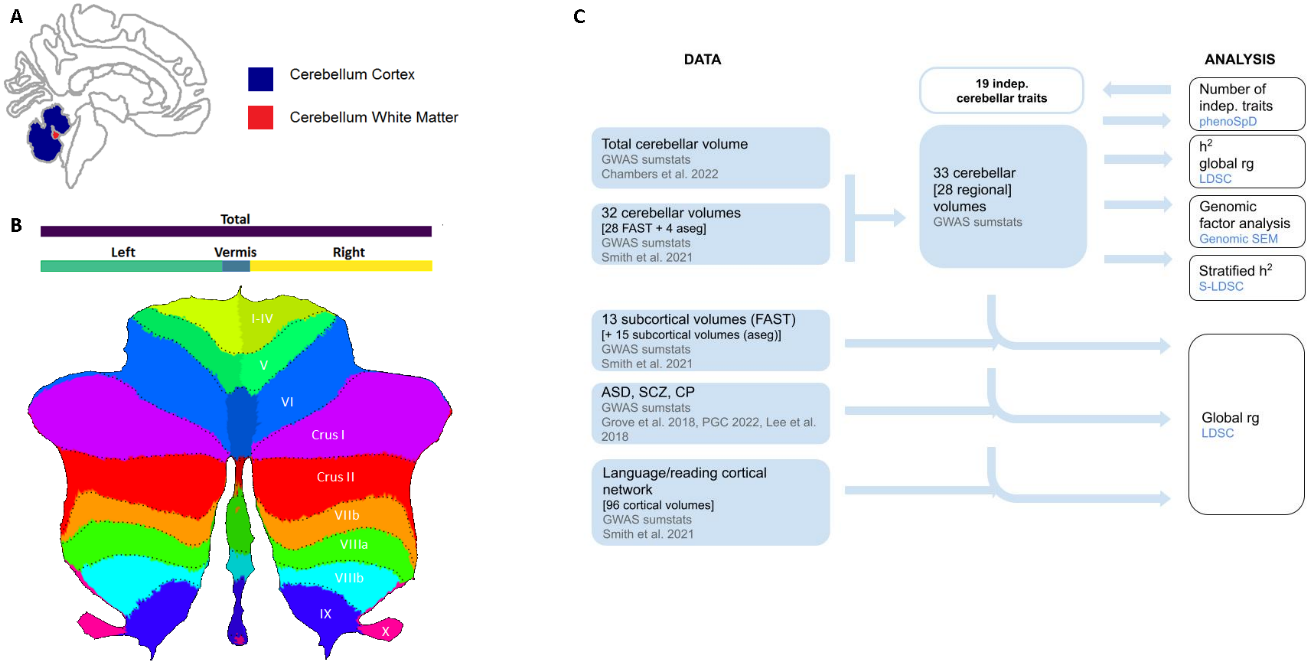

Figure S1. Cerebellar measures included in the study: **A** cerebellar cortex and white matter ('aseg' atlas, left and right), **B** total cerebellar volume4 and cerebellar lobules ('Diedrichsen atlas SUIT'[1], left, right and vermis) and **C** study workflow including datasets and analytical approach. GWAS sumstats=Genome Wide Association Study summary statistics, phenoSpD=phenotypic spectral decomposition; h^2=SNP-heritability; rg= genetic correlation; LDSC= linkage disequilibrium score regression; S-LDSC= stratified LDSC.

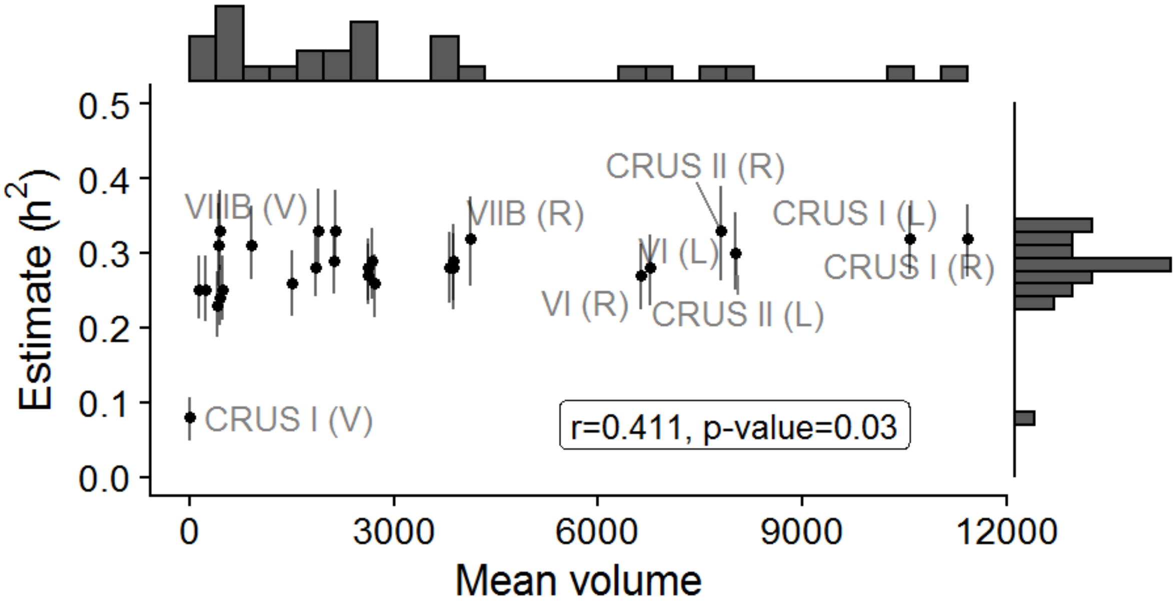

Figure S2. Heritability estimate and mean volume comparison for the cerebellar lobules. The vertical error bars indicate the 95% confidence intervals for the heritability estimate. h^2=SNP-heritability; r= Pearson's correlation coefficient.

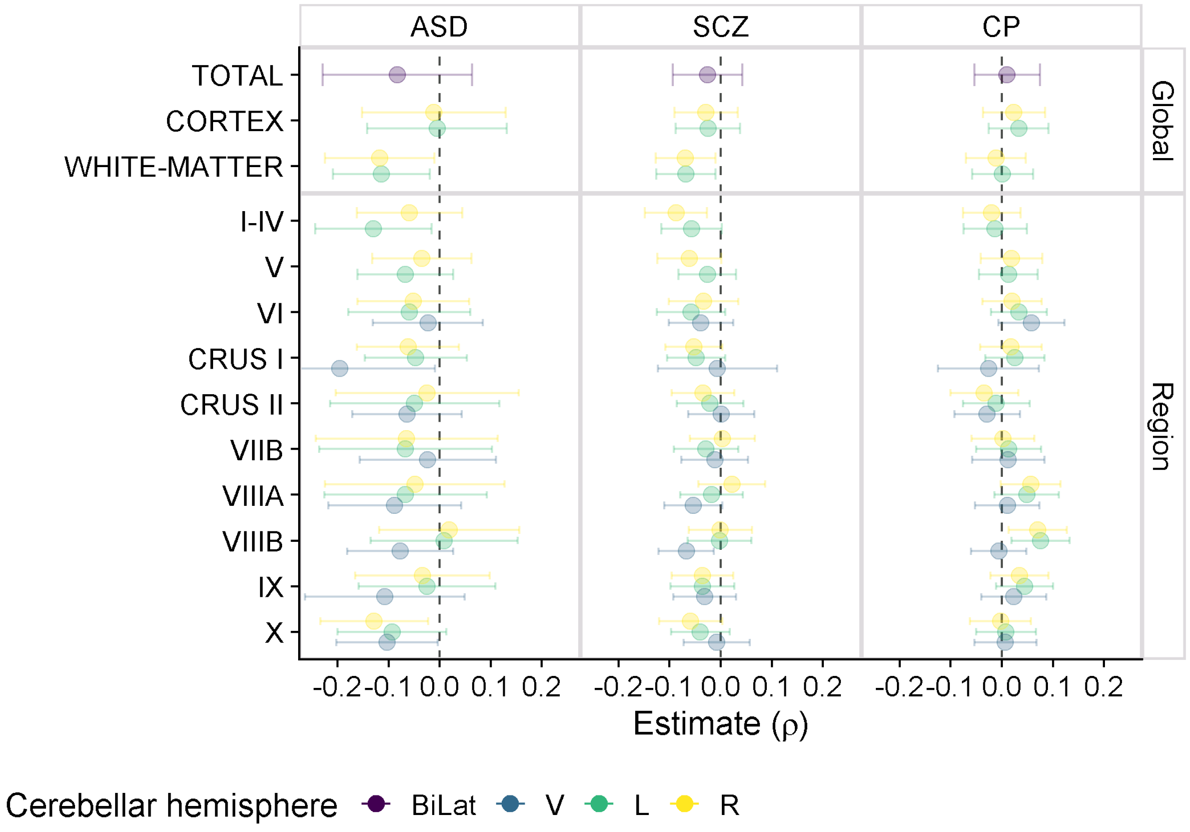

Figure S3. Global genetic correlation of cerebellar measures with psychiatric disorders and cognitive traits. Estimates are depicted by points, and error bars indicate the 95% confidence interval. All estimates had a Bonferroni corrected p-value > 0.05. ASD=Autism Spectrum Disorder; SCZ=Schizophrenia; CP=cognitive performance. Bilat=bilateral measure; V= Vermis; L= left; R=right.

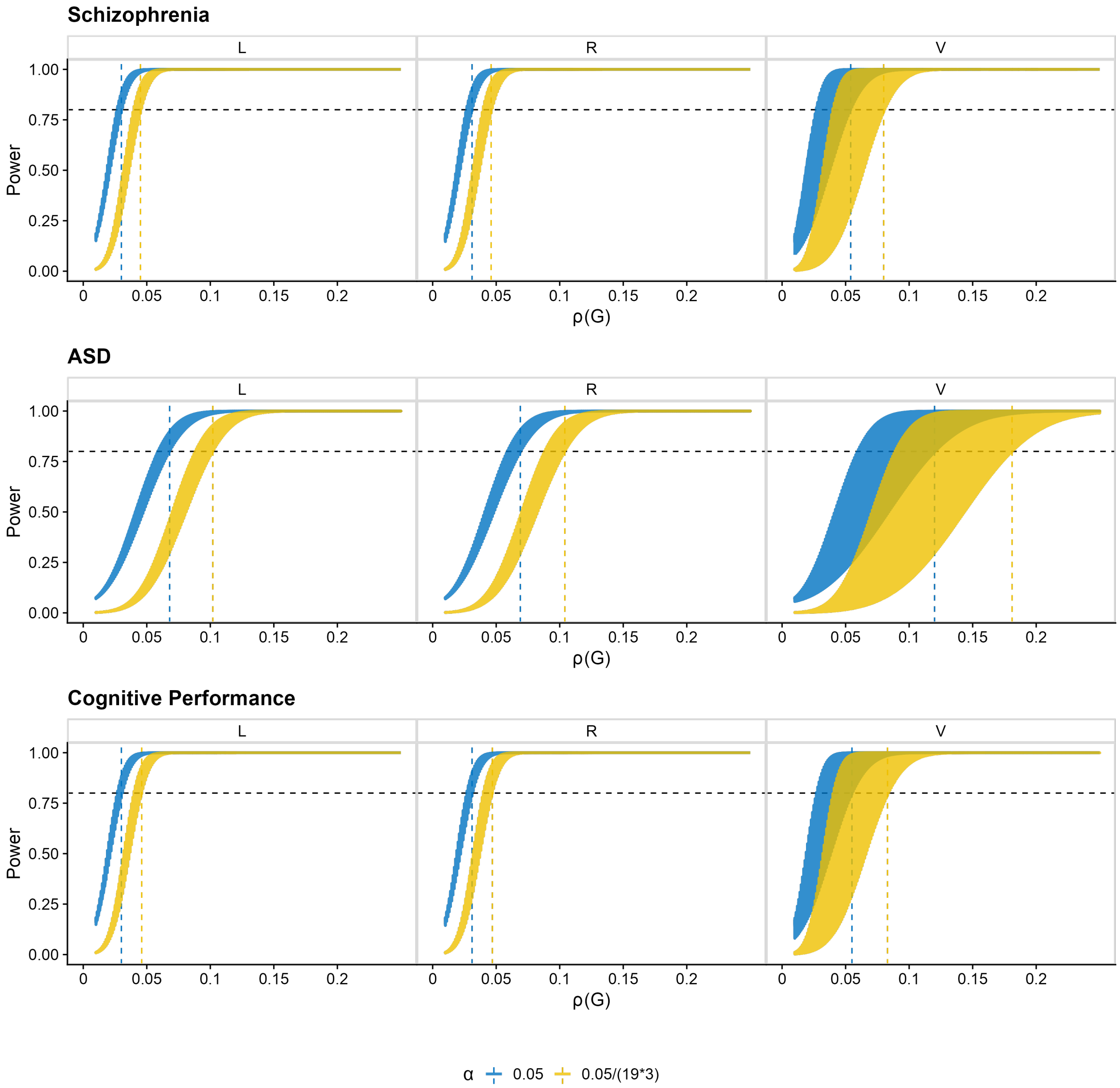

Figure S4. Approximate power to detect genetic correlation (ρ) between 33 cerebellar volume measures and psychiatric, cognitive and behavioural traits of interest (rows). The GCTA power calculator was used in all cases, into which were input different estimates of SNP-based heritability (h^2_{SNP}) that were produced by each different method for measuring genetic correlation (LDSC). See Figure 1for the h^2_{SNP} estimates for the cerebellar measures, and Table S1 for the h^2_{SNP} estimates for the other traits of interest. The dashed horizontal line indicates β=0.80. The vertical lines indicate the minimum genetic correlation (ρ value) detectable at β=0.80 and two levels of α, uncorrected 5% and Bonferroni corrected for 19$\times$3 tested genetic correlations, assuming the method-specific SNP-based heritabilites. ASD=Autism Spectrum Disorder; Bilat=bilateral measure; V= Vermis; L= left; R=right.

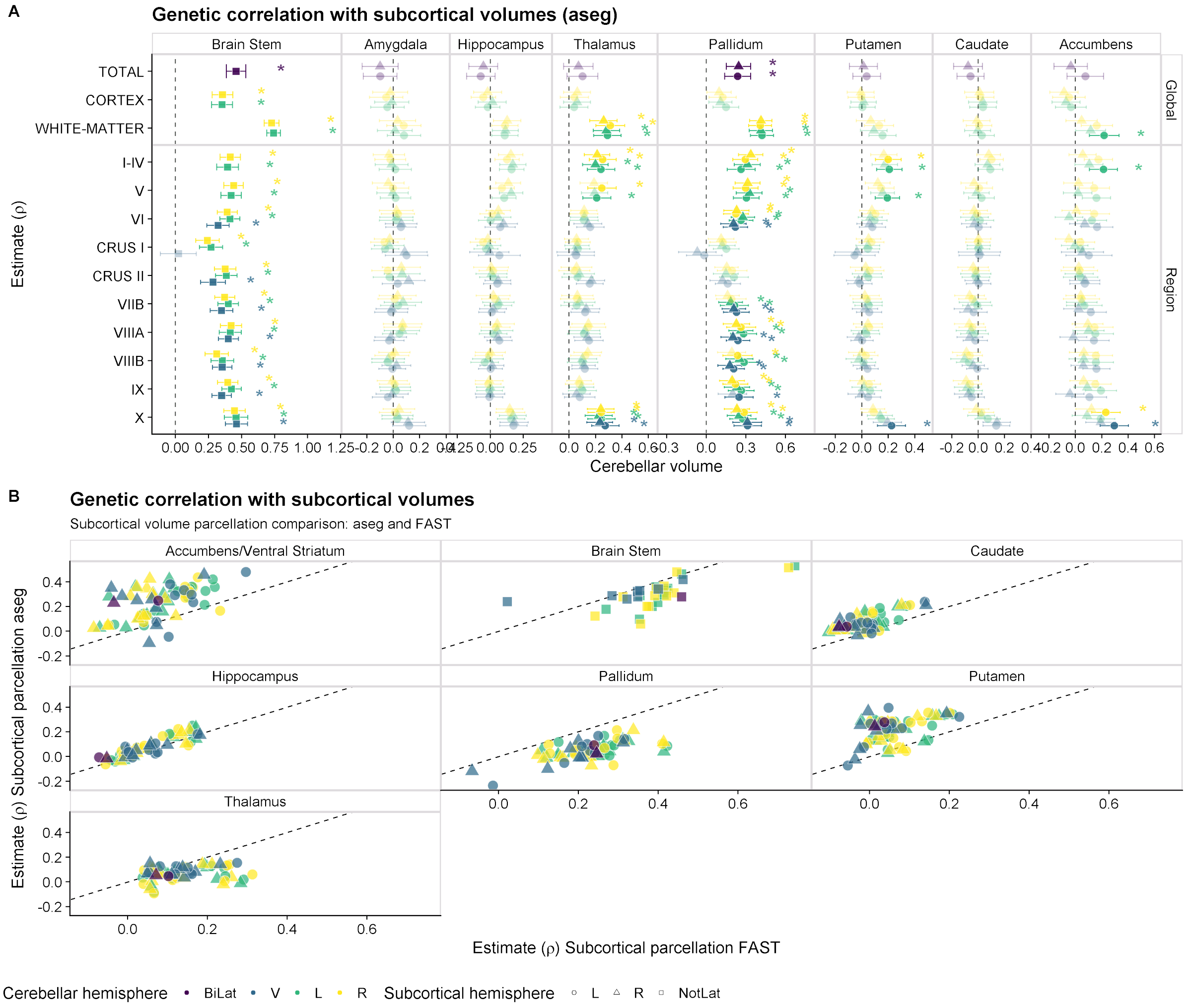

Figure S5. Genetic correlation with subcortical volumes. **A** Genetic correlation between 33 cerebellar measures with subcortical volumes derived using 'aseg' parcellation. B Comparison of the genetic correlation between cerebellar volumes and the two subcortical parcellations ('FAST' and 'aseg') for each subcortical structure.

References

1. Diedrichsen, J., Balsters, J. H., Flavell, J., Cussans, E. & Ramnani, N. A probabilistic MR atlas of the human cerebellum. *NeuroImage* **46**, 39–46, DOI: 10.1016/j.neuroimage.2009.01.045 (2009).
